# Usefulness of the Simple Coma Scale, a Simplified Version of the Glasgow Coma Scale

**DOI:** 10.1089/neur.2024.0096

**Published:** 2024-09-26

**Authors:** Soichiro Seno, Makoto Aoki, Tetsuro Kiyozumi, Kojiro Wada, Satoshi Tomura

**Affiliations:** ^1^ Department of Traumatology and Critical Care Medicine, National Defense Medical College, Tokorozawa, Japan.; ^2^ Division of Traumatology, Research Institute, National Defense Medical College, Tokorozawa, Japan.; ^3^ Department of Neurosurgery, National Defense Medical College, Tokorozawa, Japan.

**Keywords:** area under the curve, Glasgow Coma Scale, receiver operating characteristic, traumatic brain injury

## Abstract

The Glasgow Coma Scale (GCS) is the most commonly used consciousness rating scale worldwide. Although it is a sensitive and accurate way of assessing a patient’s level of consciousness, it is time-consuming and requires training. We designed the Simple Coma Scale (SCS) as a simplified version of the GCS. In this study, we examined whether the SCS could predict favorable neurogenic outcomes at discharge, survival, and GCS scores in patients with traumatic brain injury (TBI). We analyzed the data of 1,230 patients registered in the Japan Neurotrauma Data Bank (Project 2015) between April 2015 and March 2017. In the SCS, eye, verbal, and motor scores are given based on a 3-point scoring system, with similar wording (“Normal,” “Something Wrong,” and “None”) used for all scores. The SCS is based on a 7-point scale. The Glasgow Outcome Scale was used to assess the outcomes. For the receiver operating characteristic (ROC) curves with the objective variable of good prognosis at discharge in the SCS and GCS, the area under the curve (AUC) for the SCS was 0.740 (95% confidence interval [CI]: 0.711–0.769), and that of the GCS was 0.757 (95% CI: 0.729–0.786). For ROC curves with survival as the objective variable, the AUC of the SCS was 0.751 (95% CI: 0.724–0.778), and that of the GCS was 0.764 (95% CI: 0.737–0.791). The SCS, similar to the GCS, may predict good prognosis and survival at discharge. Further analyses will continue to examine the usefulness and practicality of the SCS.

## Introduction

The Glasgow Coma Scale (GCS) was developed by Teasdale et al. in 1974 to assess impairments in patient consciousness.^1^ Today, the GCS is used in >80 countries and regions and it is one of the world’s best-known awareness rating scales.^2^ However, whether the GCS is accurate and convenient is debatable.

Experience and knowledge are required for the accurate scoring of the GCS, and continuous education and training are necessary to maintain accuracy.^2^ Previous literature has reported that the accuracy of GCS scoring depends on the scorer’s job title and years of experience.^3^ Therefore, more simplified consciousness rating scales have been developed.^2,4–8^ Although more simplified GCSs have been described,^4–8^ limited consciousness rating scales exist that simplify all the GCS’s eye, verbal, and motor components.^6,8^ As the Simple Coma Scale (SCS) simplifies all items in the GCS using the same criteria and because it uses the same wording, it may be easier to score than other simplified GCSs.

Therefore, we propose an SCS that allows anyone to accurately and easily assess impairments in patient consciousness. In this study, we examined whether the SCS can predict neurological prognosis, survival, and GCS scores in patients with severe traumatic brain injury (TBI). Our hypothesis was that the SCS has comparable predictivity in predicting good prognosis at discharge and survival in patients with severe TBI.

## Methods

### Study design and participants

This study was performed using the Japan Neurotrauma Data Bank (JNTDB) and was approved by the Ethics Committee of the National Defense Medical College, Saitama, Japan (approval number: 2205). The requirement for obtaining informed consent was waived due to the anonymization of the data set used in the study. The JNTDB is a nationwide Japanese data registry designed to collect and report data on patients with TBI in Japan; it was initiated by the Japan Society of Neurotraumatology in 1996. The JNTDB typically conducts 2-year projects.^9,10^

We used data from the JNTDB Project 2015. This project was conducted from April 1, 2015, to March 31, 2017, and involved 1,345 registered patients from 32 hospitals and facilities in Japan.^10^ The criteria for enrollment in the JNTDB are TBI with a GCS ≤8 on admission, patients with TBI that worsened to a GCS ≤8 in hospital, or patients with TBI who underwent neurosurgery at the hospital.^9,10^ The exclusion criteria of the JNTDB are chronic subdural hematoma and patients with cardiopulmonary arrest on admission without an obvious cause of TBI.^9,10^ In addition to these exclusion criteria, our study excluded the following patients: (1) patients younger than 5 years; (2) patients sedated on admission; and (3) patients with missing outcomes. We excluded the children younger than 5 years because the best verbal response of “oriented” and the best motor response of “obeys commands” were not possible in those children according to Teasdale et al.^2^

### Variables collected for analysis

The JNTDB data sheet comprised >200 items, including information on the characteristics of the injury and diagnosis, treatment, and complications concerning treatment outcome.^9^ The collected variables were age, sex, total GCS score, mechanism, antithrombotic medication, alcohol consumption, transportation, Traumatic Coma Data Bank (TCDB) classification,^11^ injury severity score, Abbreviated Injury Scale (AIS) of the head, multiple trauma, hospitalization period, vital signs on admission, pupil dilation, initial computed tomography (CT) findings, neurosurgical intervention, and blood coagulation examination. Multiple trauma was defined as severe trauma with an AIS ≧ 3 in addition to that sustained in the head region.

### Study outcomes

The primary outcome was evaluated using the Glasgow Outcome Scale (GOS) at discharge. Favorable outcomes included good recovery and moderate disability. Poor outcomes included severe disability, vegetative state, and death.^12^ The secondary outcome was in-hospital survival.

### SCS

The SCS is a simplified version of the GCS. The lowest score for each item in the GCS (E:1, V:1, M:1) was 1 point in the SCS, the highest score for each item (E:4, V:5, M:6) was 3 points in the SCS, and all other items (E:2–3, V:2–4, M:2–5) were scored 2 points in the SCS. Consequently, the GCS, which had a 13-point scale (minimum score of 3, total score of 15), was reduced to a 7-point scale (minimum score of 3, total score of 9) in the SCS. Three points for each item on the SCS indicated normal conditions with no abnormalities. If something was amiss, the item would be given a score of 2. An SCS score of 1 indicated that there was no response. In other words, 3 points in the SCS were given for “Normal,” 1 point for “None,” and 2 points for “Something Wrong” (Table 1).

**Table 1.** Simple Coma Scale Scoring

	Simple Coma Scale		
	3	2	1
Eye	Normal	Something Wrong	None
Verbal	Normal	Something Wrong	None
Motor	Normal	Something Wrong	None

### Statistical analysis

Descriptive statistics were calculated for all variables. Continuous variables were presented as medians and interquartile ranges (IQRs), and categorical variables as percentages and numbers.

Receiver operating characteristic (ROC) curves were created to show the predictivity of the SCS and GCS scores for the outcomes. The area under the receiver-operating-characteristic curve (AUROC) and the associated 95% confidence intervals (CIs) were calculated^13^ and compared between the SCS and GCS scores. AUROCs were also calculated for the Eye alone, Verbal alone, and Motor alone subscales of the SCS. A clinically significant difference in the AUROC was defined *a priori* as ≥0.05 based on previous literature.^5,14^ Box-and-whisker plots were created to clarify the relationship between the SCS and GCS scores. R version 4.3.2 (The R Foundation for Statistical Computing, Vienna, Austria) was used for statistical analysis.

## Results

**FIG. 1.**
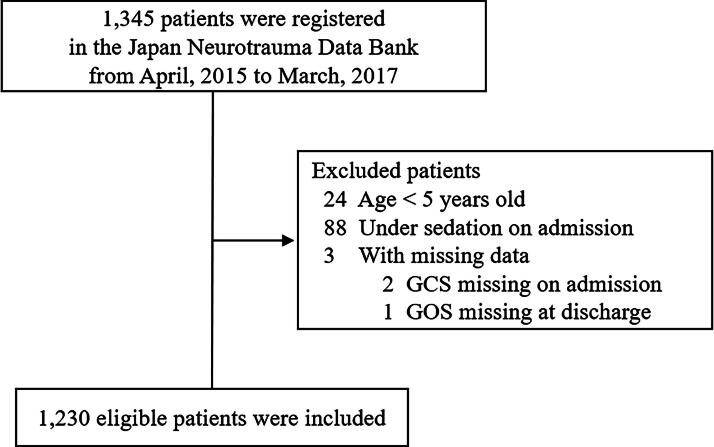
Flowchart of the study design.

Of the 1,345 patients enrolled in the JNTDB Project 2015, 1,230 were included in the analysis (Fig. 1). The characteristics of the study participants are presented in Table 2. The median age was 67 (44–79) years, and 30.5% were women. The median total GCS score was 6 (4–10). Traffic and nontraffic accidents were equally prevalent in terms of the origin of injury. Approximately 12% of the patients were taking antiplatelet medication and 8% were taking anticoagulant medication. JNTDB classified the patients according to the TCDB classification, with diffuse injury in 31.9% and mass lesions in 68.0% of patients. Isolated TBIs were present in 59.0% of patients and multiple traumas in 41.0%. During hospitalization, 66.9% of the patients underwent neurosurgery (Table 2). Patient characteristics, except for those shown in Table 2, are shown in Supplementary Table S1.

**Table 2.** Patient Characteristics

Variables	*n* = 1,230
Age, years, median (IQR)	67 (44–79)
Age category, years	
5–18	91 (7.4)
19–64	461 (37.5)
65–84	544 (44.2)
≥85	134 (10.9)
Sex, female	375 (30.5)
GCS (Total score) (IQR)	7 (4–11)
Mechanism	
Traffic accident	505 (41.1)
Nontraffic accident	
Fall from height	308 (25.0)
Fall from standing	315 (25.6)
Fall during sports	4 (0.3)
Nonfall sports injury	5 (0.4)
Other mechanism	87 (7.1)
Unknown	6 (0.5)
Antiplatelet medication	155 (12.6)
Anticoagulant medication	105 (8.5)
TCDB classification	
Diffuse injury	392 (31.9)
Mass lesion	837 (68.0)
Unknown	1 (0.0)
Injury severity score (IQR)	25 (16–30)
AIS of head	
1	49 (4.0)
2	31 (2.5)
3	119 (9.7)
4	308 (25.0)
5	620 (50.4)
6	42 (3.4)
Unknown	61 (5.0)
Multiple trauma	
Isolated TBI	726 (59.0)
Multiple trauma with TBI	504 (41.0)
Neurosurgery	823 (66.9)

IQR; interquartile range, GCS; Glasgow Coma Scale, TCDB classification; Traumatic Coma Data Bank classification, AIS; Abbreviated Injury Scale, TBI; traumatic brain injury.

**FIG. 2.**
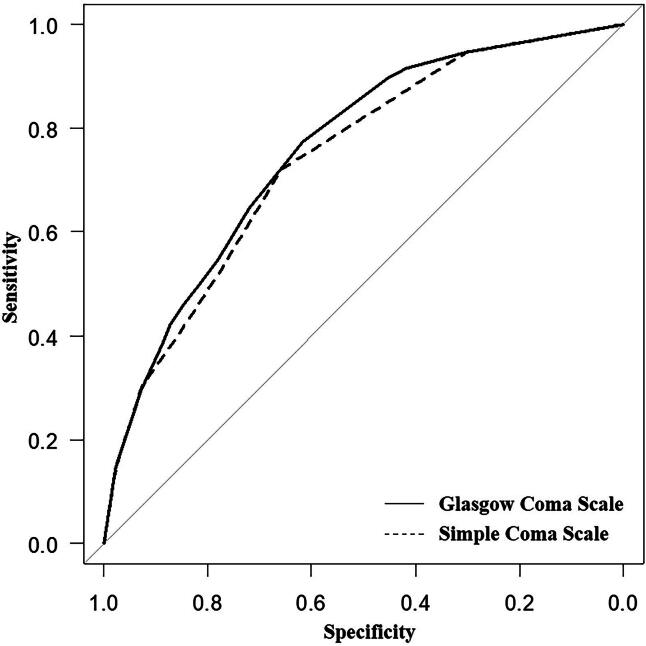
Receiver operator characteristic (ROC) curve for prediction of outcomes at discharge. For the ROC curves with the objective variable of good prognosis at discharge in SCS and GCS, the area under the curve (AUC) for SCS was 0.740 (95% CI: 0.711–0.769) and that for GCS was 0.757 (95% CI: 0.729–0.786). SCS, Simple Coma Scale; GCS, Glasgow Coma Scale; ROC, receiver operating characteristic; AUC, area under the curve; CI, confidence interval.

**FIG. 3.**
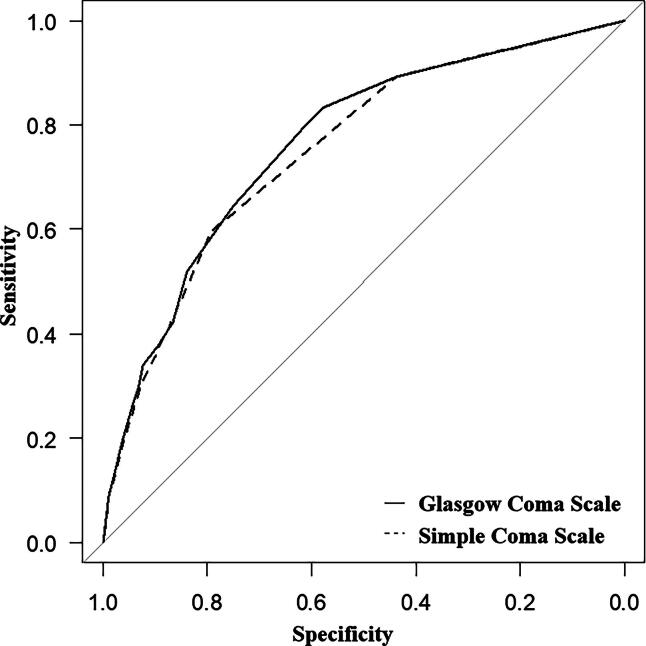
Receiver operator characteristic (ROC) curve for prediction of survival at discharge. For ROC curves with survival as the objective variable, the area under the curve (AUC) of SCS was 0.751 (95% CI]: 0.724–0.778), and that for GCS was 0.764 (95% CI: 0.737–0.791). SCS, Simple Coma Scale; GCS, Glasgow Coma Scale; ROC, receiver operating characteristic; AUC, area under the curve; CI, confidence interval.

Regarding the ROC curves for favorable outcomes, the area under the curve (AUC) of SCS and GCS was 0.740 (95% CI: 0.711–0.769) and 0.757 (95% CI: 0.729–0.786), respectively (Fig. 2). No significant difference was observed in the AUC for favorable outcomes between the SCS and GCS scores. Table 3 shows the actual distribution of patient GOS scores at discharge using the SCS and GCS scores. On the ROC curves for survival, the AUC of SCS and GCS was 0.751 (95% CI: 0.724–0.778) and 0.764 (95% CI: 0.737–0.791), respectively (Fig. 3). The AUC for survival of the SCS and GCS did not differ significantly.

**Table 3.** Distribution of Patient Glasgow Outcome Scale Scores at Discharge Using the Simple Coma Scale and Glasgow Coma Scale

SCS	3	4	5	6	7	8	9							
GR	3	31	34	18	17	38	36							
MD	16	50	38	24	18	19	16							
SD	32	97	45	22	40	23	10							
VS	32	50	24	21	6	7	5							
D	200	163	37	24	15	15	4							

GCS	3	4	5	6	7	8	9	10	11	12	13	14	15	
GR	3	3	4	14	18	21	7	6	8	7	12	38	36	
MD	16	8	3	22	28	17	15	6	9	7	18	16	16	
SD	32	20	10	53	36	22	11	13	11	5	23	23	10	
VS	32	15	6	34	15	16	3	5	2	2	3	7	5	
D	200	65	14	65	40	11	13	11	6	7	9	13	4	

SCS, Simple Coma Scale; GCS, Glasgow Coma Scale; GR, good recovery; MD, moderate disability; SD, severe disability, VS, vegetative state; D; death.

**FIG. 4.**
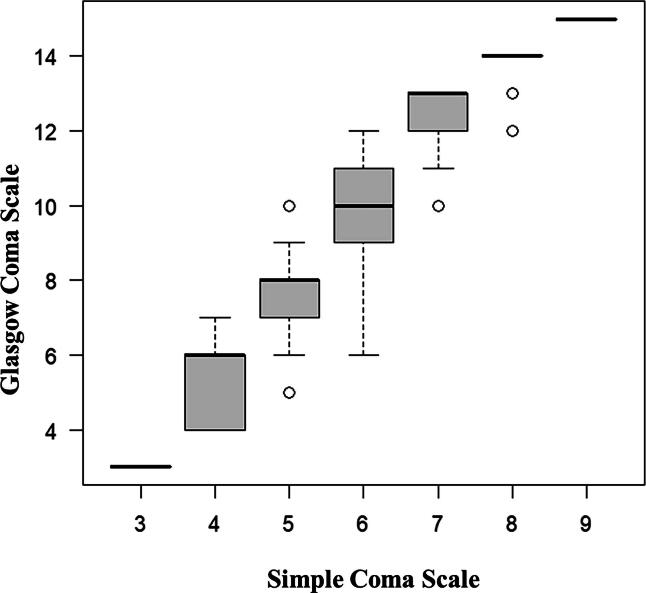
Box plots to visualize the relationship between the Simple Coma Scale and Glasgow Coma Scale. Lines in the box represent median values, and box edges represent 25th to 75th percentiles. Lines extending outside the box indicate 10th and 90th percentiles, respectively. Circles indicate outliers.

Figure 4 shows box plots visualizing the relationship between the SCS and GCS scores. Table 4 shows the conversion of SCS to GCS.

**Table 4.** Developed Conversion Table of the Simple Coma Scale to the Glasgow Coma Scale

Simple Coma Scale	Glasgow Coma Scale
3	3
4	6
5	8
6	10
7	13
8	14
9	15

## Discussion

In this study, we introduced the SCS, a simplified version of the GCS, which has comparable predictivity in predicting good prognosis at discharge and survival in patients with severe TBI (Figs. 2, 3).

Several simplified GCSs have been previously described.^4–8^ Gill et al. proposed the Simplified Motor Scale and the Simplified Verbal Scale and compared their validity with that of the GCS.^5^ The Simplified Motor Scale consists only of motor items, and it is helpful in prehospital settings.^7^ The score that Bodien et al. developed is divided into six levels.^8^ They assessed the verbal characteristics first, followed by the motor characteristics.^8^ Few consciousness rating scales exist that simplify all the GCS’s eye, verbal, and motor components.^6,8^ This SCS may be easier to score than other simplified GCSs because it simplifies all items in the GCS using the same criteria and it uses the same wording. As shown in Supplementary Table S4, the AUC of the SCS was comparable with that of the other simplified GCSs; however, the AUCs of the Eye alone, Verbal alone, and Motor alone subscales of the SCS were inferior to those of the SCS. In this study, we included patients with severe TBI who had GCS ≤8 on admission or in hospital, or who subsequently underwent neurosurgery in hospital. They included those whose GCS total scores on admission were ≥9. It is necessary to verify whether SCS can achieve comparable results if it targets all TBIs, as in other simplified GCSs.^5,7,8^

Severe TBI is usually defined as a GCS score of ≤8.^15^ In Table 4, a GCS score of 8 corresponds to an SCS score of 5. As shown in Supplementary Table S2, the proportions of patients with a GCS score of ≤8 and an SCS score ≤5 are similar, and the prognosis at discharge is also similar. In the box-and-whisker plots in Figure 4, the SCS score of 6 varied much more than the other scores. Some severe TBIs defined as GCS ≤8 are also included in the SCS score ≤6 (11.9%), and TBIs not defined as severe TBIs with GCS ≥9 are included in the SCS score ≥5 (12.4%) (Supplementary Table S3). Further discussion and validation are required to define severe TBI that is SCS ≤5, which is equivalent to GCS ≤8.

The most significant advantage of the SCS over the GCS is its apparent simplicity; each of the E, V, and M items is identified using the same word. We believe that anyone can learn how to score the SCS and use it in clinical practice. This may be beneficial for doctors who are not accustomed to assessing consciousness and for nurses and paramedics who are less confident in their knowledge of neurology. In future, it will be necessary to verify whether the SCS scoring yields similar results if people with different job titles and years of experience assess for consciousness using the SCS.

The disadvantage of this simplification is that it is difficult to capture minor changes in patients. Patients with TBI are considered to “talk and then deteriorate” if their GCS score declines after admission.^16^ Close monitoring of GCS scores over time leads to the clinical suggestion that intracranial lesions should be reevaluated, for example, using head CT.^16,17^ When providing intensive care in the intensive care unit (ICU), it is necessary to observe changes in consciousness and respiratory status over time in detail. The FOUR score, which observes brainstem reflexes and respiration patterns in addition to visual and motor characteristics, is more suitable for detailed patient observation in ICUs.^18^ This SCS may not be appropriate when the evaluator wants to observe changes in the patient’s condition in detail and over time. However, with proper training and an understanding of its limitations, the SCS may still be a valuable tool for assessing consciousness quickly, especially in settings where prehospital, emergency medicine, mass casualties, and disasters occur. Nevertheless, it is necessary to evaluate the SCS in those actual clinical settings to verify its ease or difficulty of use.

## Limitations

In this study, patients with severe TBI or those requiring neurosurgery were analyzed. Future validation is needed to determine whether SCS can predict prognosis at discharge, survival, and the GCS score, in all TBIs or other traumas. The JNTDB Project 2015 was used in this study; it included 1,345 patients enrolled between 2015 and 2017. Therefore, the data used in this study were old and the sample size was small. Because most patients enrolled in the JNTDB were Japanese, our results may not be generalizable to other countries or ethnic groups. Future studies should be conducted on patients with TBI other than severe TBI in different countries and regions.

## Conclusion

We developed the SCS as a simplified version of the GCS. This study suggests that the SCS has comparable predictivity to the GCS in predicting good prognosis at discharge and survival in patients with severe TBI.

## Abbreviations Used

GCS: Glasgow Coma Scale
SCS: Simple Coma Scale
TBI: Traumatic Brain Injury
JNTDB: Japan Neurotrauma Data Bank
TCDB: Traumatic Coma Data Bank
AIS: Abbreviated Injury Scale
CT: Computed Tomography
GOS: Glasgow Outcome Scale
IQR: Interquartile range
ROC: Receiver Operating Characteristic
AUROC: Area Under the Receiver-Operating-Characteristic curve
CI: Confidence Interval
AUC: Area Under the Curve
ICU: Intensive Care Unit
GR: Good Recovery
MD: Moderate Disability
SD: Severe Disability
VS: Vegetative State
D: Death
