# Supplementary material for: Usefulness of the Simple Coma Scale, a Simplified Version of the Glasgow Coma Scale
Source: Neurotrauma Rep. 2024 Sep 26;5(1):883–9. doi: 10.1089/neur.2024.0096 (PMC11512088; doi:10.1089/neur.2024.0096)
Supplement: Supplementary Table S1 [file neur.2024.0096_s._seno_supplemental_data_2.pdf]

Supplementary Table 1. Patient characteristics (except for those shown in Table 2.)

**Variables**	n = 1,230
Drinking alcohol	
Yes	172 (14.0)
No	867 (70.5)
unknown	191 (15.5)
Transportation	
Ambulance	1144 (93.0)
Helicopter	67 (5.4)
Car	5 (0.4)
On foot	9 (0.7)
Unknown	5 (0.4)
TCDB classification	
Diffuse injury I	35 (2.8)
Diffuse injury II	241 (19.6)
Diffuse injury III	82 (6.7)
Diffuse injury IV	34 (2.8)
Evacuated mass lesion	473 (38.5)
Non-evacuated mass lesion	364 (29.6)
Unknown	1 (0.0)
Hospitalization period (day)*	19 (3-41)
Vital signs on admission	
Systolic blood pressure (mmHg)**	146.2 ± 38.4
Heart rate (/min)**	91.2 ± 24.5
Respiratory rate (/min)**	21 ± 7
Temperature (° C)**	36.2 ± 1.3
Pupil dilation	
Both sides	270 (22.0)
One side	181 (14.7)
None	762 (62.0)
Unknown	17 (1.4)

* IQR

** Standard Deviation

Abbreviations: IQR; Interquartile range, TCDB classification; Traumatic Coma Data Bank classification

Supplementary Table 1. (continued) Patient characteristics (except for those shown in Table 2.)

**Variables**	n = 1,230
Initial CT findings	
Fornix fracture	599 (48.7)
Skull base fracture	364 (29.6)
Pneumoencephaly	274 (22.3)
Subarachnoid hemorrhage	886 (72.0)
Intraventricular hemorrhage	213 (17.3)
Posterior fossa local lesion	79 (6.4)
Midline shift* (mm)	2.0 (0.0-10.9)
Hematoma types	
Acute subdural hematoma***	613 (49.8)
Acute epidural hematoma***	149 (12.1)
Intracerebral hematoma***	74 (6.0)
Contusion***	142 (11.5)
Neurosurgical intervention	
ICP monitoring***	354 (28.8)
External ventricular drainage***	66 (5.4)
Hematoma removal by perforation***	224 (18.2)
Hematoma removal by craniectomy***	222 (18.0)
Decompressive craniectomy***	254 (20.7)
Blood coagulation examination	
Platelet*	19.6 (14.8-25.8)
PT-INR*	1.10 (1.02-1.24)
APTT*	29.5 (26.1-35.2)
Fibrinogen*	241 (179-307)
D-dimer*	36.2 (12.2-94.2)

* IQR

***There is some overlapping

Abbreviations: IQR; Interquartile range, CT; Computed Tomography, ICP; Intracranial Pressure, PT-INR; Prothrombin Time-International Normalized Ratio, APTT; Activated Partial Thromboplastin Time

Supplementary Table 2. The outcomes at discharge for SCS ≤ 5 and GCS ≤ 8.

	SCS ≤ 5	GCS ≤ 8
GR	68	63
MD	104	94
SD	174	173
VS	106	118
D	400	395
Total	852	843

Abbreviations: SCS; Simple Coma Scale, GCS; Glasgow Coma Scale, GR; good recovery, MD; moderate disability, SD; severe disability, VS; vegetative state, D; death

Supplementary Table 3. Total GCS score and each SCS subscore in SCS 5 and SCS 6.

SCS 5

GCS total score	SCS	n = 178
GCS 5	SE:1 SV:2 SM:2	5 (2.8)
	SE:2 SV:1 SM:2	1 (0.1)
	SE:2 SV:2 SM:1	1 (0.1)
GCS 6	SE:1 SV:2 SM:2	9 (5.1)
	SE:2 SV:1 SM:2	1 (0.1)
	SE:3 SV:1 SM:1	8 (4.5)
GCS 7	SE:1 SV:2 SM:2	47 (26.4)
	SE:2 SV:1 SM:2	8 (4.5)
GCS 8	SE:1 SV:2 SM:2	67 (37.6)
	SE:2 SV:1 SM:2	9 (5.1)
GCS 9	SE:1 SV:2 SM:2	11 (6.2)
	SE:2 SV:1 SM:2	10 (5.6)
GCS 10	SE:1 SV:2 SM:2	1 (0.1)

SCS 6

GCS total score	SCS	n = 109
GCS 6	SE:2 SV:2 SM:2	1 (0.9)
GCS 7	SE:3 SV:2 SM:1	1 (0.9)
GCS 8	SE:2 SV:2 SM:2	10 (9.2)
	SE:3 SV:2 SM:1	1 (0.1)
GCS 9	SE:1 SV:2 SM:3	3 (2.8)
	SE:2 SV:2 SM:2	16 (14.7)
	SE:3 SV:1 SM:2	9 (8.3)
GCS 10	SE:2 SV:1 SM:3	5 (4.6)
	SE:2 SV:2 SM:2	21 (19.3)
	SE:3 SV:1 SM:2	10 (9.2)
GCS 11	SE:1 SV:2 SM:3	2 (1.8)
	SE:1 SV:3 SM:2	1 (0.1)
	SE:2 SV:2 SM:2	14 (12.8)
GCS 12	SE:2 SV:2 SM:2	15 (13.8)

Abbreviations: SCS; Simple Coma Scale, GCS; Glasgow Coma Scale, SE; Eye in Simple Coma Scale, SV; Verbal in Simple Coma Scale, SM; Motor in Simple Coma Scale

Supplementary Table 4. Comparison of the Simplified GCSs.

Authors	Scale (Name)	Patients	n	Outcome	AUROC for the scale	AUROC for the GCS
Seno et al.	Simple Coma Scale (SCS)	severe TBI	1,230	Glasgow Outcome Scale	0.74 (95% CI: 0.71 – 0.77)	0.76 (95% CI: 0.73 – 0.79)
	Eye alone of SCS				0.61 (95% CI: 0.57 – 0.65)	
	Verbal alone of SCS				0.63 (95% CI: 0.61 – 0.66)	
	Motor alone of SCS				0.60 (95% CI: 0.57 – 0.62)	
	Simple Coma Scale (SCS)			Mortality	0.75 (95% CI: 0.72 – 0.78)	0.76 (95% CI: 0.74 – 0.79)
	Eye alone of SCS				0.66 (95% CI: 0.63 – 0.69)	
	Verbal alone of SCS				0.66 (95% CI: 0.63 – 0.68)	
	Motor alone of SCS				0.68 (95% CI: 0.64 – 0.71)	
Thompson et al.	Simplified Motor Score	all TBI	1,125	Mortality	0.74 (95% CI: 0.70 – 0.77)	0.82 (95% CI: 0.74 – 0.90)
	Eye alone of GCS				0.79 (95% CI: 0.71 – 0.86)	
	Verbal alone of GCS				0.78 (95% CI: 0.71 – 0.85)	
	Motor alone of GCS				0.76 (95% CI: 0.70 – 0.83)	
Gill et al.	Simplified Verbal Scale	all TBI	7,160	Mortality	0.84 (95% CI: 0.85 – 0.88)	0.89 (95% CI: 0.88 – 0.90)
	Simplified Motor Scale				0.86 (95% CI: 0.86 – 0.89)	
	Eye alone of GCS				0.85 (95% CI: 0.83 – 0.86)	
	Verbal alone of GCS				0.87 (95% CI: 0.85 – 0.88)	
	Motor alone of GCS				0.88 (95% CI: 0.87 – 0.89)	

Abbreviations: SCS; Simple Coma Scale, GCS; Glasgow Coma Scale, TBI; traumatic brain injury, AUROC; area Under the Receiver-Operating-Characteristic curve, CI; Confidence Interval
